# Dual-Mode Radar Sensor for Indoor Environment Mapping

**DOI:** 10.3390/s21072469

**Published:** 2021-04-02

**Authors:** Seongwook Lee, Song-Yi Kwon, Bong-Jun Kim, Hae-Seung Lim, Jae-Eun Lee

**Affiliations:** 1 School of Electronics and Information Engineering, College of Engineering, Korea Aerospace University, Gyeonggi-do 10540, Korea; thddl1622@kau.kr; 2 Bitsensing Inc., Seoul 06247, Korea; bongjun@bitsensing.com (B.-J.K.); lim.h@bitsensing.com (H.-S.L.); jlee@bitsensing.com (J.-E.L.)

**Keywords:** dual-mode detection, frequency-modulated continuous wave radar, multiple-input multiple-output antenna, simultaneous localization and mapping

## Abstract

In this paper, we introduce mapping results in an indoor environment based on our own developed dual-mode radar sensor. Our radar system uses a frequency-modulated continuous wave (FMCW) with a center frequency of 62 GHz and a multiple-input multiple-output antenna system. In addition, the FMCW radar sensor we designed is capable of dual-mode detection, which alternately transmits two waveforms using different bandwidths within one frame. The first waveform is for long-range detection, and the second waveform is for short-range detection. This radar system is mounted on a small robot that moves in indoor environments such as rooms or hallways, and the radar and the robot send and receive necessary information to each other. The radar estimates the distance, velocity, and angle information of targets around the radar-equipped robot. Then, the radar receives information about the robot’s motion from the robot, such as its speed and rotation angle. Finally, by combining the motion information and the detection results, the radar-equipped robot maps the indoor environment while finding its own position. Compared to the actual map data, the radar-based mapping is effectively achieved through the radar system we developed.

## 1. Introduction

Recently, not only the autonomous driving of automobiles, but also the autonomous driving of robots indoors is attracting a lot of attention from people. The core technology for autonomous driving of robots is simultaneous localization and mapping (SLAM), which maps the surrounding environment while the robot locates itself. The SLAM in robots has been implemented based on data acquired from laser and camera sensors mounted on a robot. However, these sensors have very poor detection performance in environments where the amount of light is insufficient or in foggy environments.

Therefore, in recent years, studies to utilize radar sensors for the mapping have been conducted because radar sensors have the advantage of less degradation in detection performance even with environmental changes. For example, indoor mapping results using ultra-wideband (UWB) radar systems were introduced in [1,2,3,4]. In [2], the mapping was performed using the acquired data while manually rotating a single UWB radar sensor in 45 degree increments. In addition, to find the angle information of the object for the SLAM, a method of sequentially transmitting UWB pulses with three different center frequencies was proposed in [3]. Recently, the authors in [4] proposed a method for obtaining spatial information of objects by using two receiving antennas in different directions. In [5], the SLAM was performed in an indoor environment using a frequency-modulated continuous wave (FMCW) radar using 24 GHz as the center frequency. In this study, the antenna was mechanically rotated 360 degrees to detect multiple targets at the same time. Moreover, the authors in [6,7] presented the mapping results in indoor smoky situations. In both studies, a radar system developed by Fraunhofer Institute for High Frequency Physics and Radar Techniques (FHR) that mechanically rotates the antenna through a motor was applied. In [8], indoor SLAM was performed using a radar sensor using the 122 GHz, but the maximum detectable distance of the radar was only a few meters.

As such, most of the radar systems used in the conventional studies on the radar-based mapping use a low frequency band, so the range resolution is inevitably low. In addition, in such studies, the angular resolution was low using a small number of antenna elements, and a method of mechanically rotating the antenna was used to overcome a narrow angle detection range. Unlike previous studies, the radar sensor we developed performs the mapping with high range and angle resolution, which does not require mechanical or manual rotation of the antenna system. The radar system we developed transmits the FMCW radar signal with a center frequency of 62 GHz. In other words, the radar can use a wider bandwidth and thus has better range resolution [9]. In addition, the FMCW radar signal has the advantage of being able to achieve high range resolution through pulse compression [10]. Moreover, instead of using a motor to rotate the antenna mechanically [5,6,7,8], our radar system adopts an electronic scanning method that uses an array antenna system. To improve the angular resolution within limited radar hardware, we also use a multiple-input multiple output (MIMO) antenna system [11]. The angular resolution is further improved by using the array interpolation method in [12] together.

The radar sensor we developed has the following advantages. The use of very short wavelengths in millimeters can reduce the size of the antenna system, allowing the radar to be miniaturized. In addition, compared to radar systems using the UWB (i.e., 3.1 to 10.6 GHz), our radar system adopting the millimeter wave band can use a wider bandwidth and thus has better range resolution. Moreover, unlike the mechanical scanning methods, the electronic scanning method has the advantage that it can be operated in several modes simultaneously and has a short detection cycle [13]. As such, despite its small size compared to the conventional radar systems, our radar sensor has superior range and angular resolution and is suitable for high-resolution object detection in an indoor environment. When the radar detects the position of an object in high resolution, the mapping result of the indoor environment becomes more accurate.

We mount this radar sensor on a small robot and conduct measurements in indoor corridor environments. To perform the mapping using a radar sensor, it does not end with plotting the radar detection result, but continuously reflecting the movement of the robot. Thus, the radar system receives information about the moving velocity and rotation angle of the robot from its motor and the radar detection result is corrected by considering the robot’s movement every frame. Finally, the mapping is achieved by accumulating the corrected detection results and a map around the radar-equipped robot is plotted. In the process of performing the mapping, moving targets can hinder the radar sensor from drawing an accurate map. Thus, we improve the accuracy of the mapping result by removing moving targets in the mapping result. One of the advantages of the radar-based mapping is that the radar can immediately discriminate between moving and stationary targets based on their estimated velocities because the velocity of the target can be estimated directly by applying Fourier analysis [14]. However, unlike radar sensors, because the main purpose of camera sensors or lidar sensors is to extract an image of a target, additional signal processing algorithms [15] or waveform changes [16] are required to estimate the velocity information of the target from those sensors.

The remainder of this paper is organized as follows. First, the dual-mode FMCW radar system that we developed is introduced in Section 2. Then, in Section 3, a method of estimating the distance, velocity, and angle of the target from the obtained radar sensor data is explained. In addition, a method of converting the radar detection result into the two-dimensional (2D) range map and combining the detection results of the two modes is introduced. In Section 4, we describe the experimental environment in which the radar-equipped robot is driving and show the mapping results in that environment. Finally, we conclude this paper in Section 5.

## 2. Dual-Mode FMCW Radar Sensor

**Figure 1:**
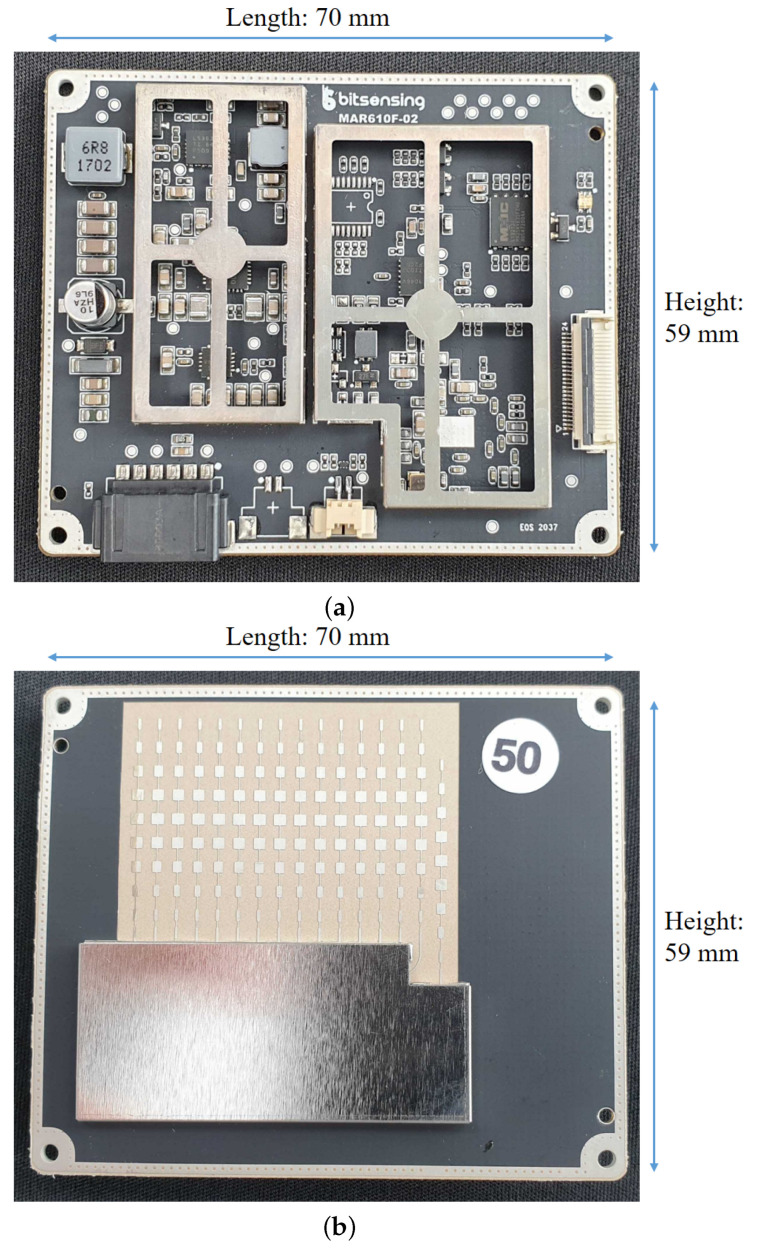
Assembled PCB of the dual-mode FMCW radar system: (**a**) the front side; (**b**) the back side.

For target detection in indoor environments, we developed the dual-mode FMCW radar system that operates in the millimeter wave band. In this study, the dual-mode means that both long-range detection and short-range detection are possible with a single radar sensor. Figure 1 shows the front and back sides of the assembled printed circuit board (PCB) of the radar system. The length, height, and width of the assembled PCB are 70 mm, 59 mm, and 9 mm, respectively. As shown in Figure 1b, a patch antenna system was used, which consists of several transmit and receiving antenna elements. Depending on the long-range detection mode or the short-range detection mode, different transmit and receiving antenna elements are selected and used.

**Figure 2:**
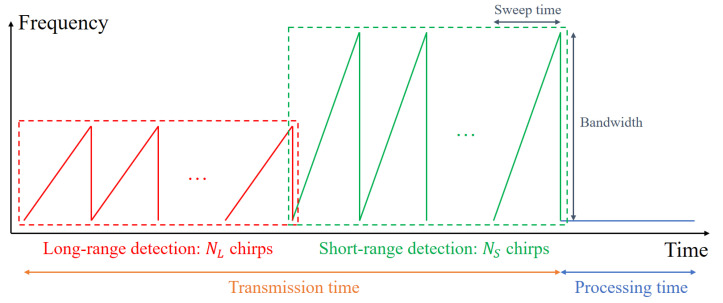
FMCW radar signal corresponding to one frame.

Figure 2 shows the time-frequency slope of the FMCW radar signal transmitted at each frame in our radar system. One frame defined in this study is the basic time unit of radar signal processing, which consists of signal transmission time and signal processing time, and its total length is 50 ms. As shown in Figure 2, multiple waveforms whose frequency increases linearly with time are transmitted sequentially within the transmission time. To increase the range resolution while miniaturizing the antenna system of the radar, the millimeter wave band was used. In addition, waveforms with a relatively narrow bandwidth are used for long-range detection, whereas waveforms with a relatively wide bandwidth are used for short-range detection. In our radar system, based on the center frequency ($f_{c}$) of 62 GHz, 1.5 GHz and 3 GHz are used as bandwidths for long-range detection and short-range detection, respectively. In general, the range resolution Δr of the FMCW radar signal is determined by $\Delta r=\frac{c}{2B}$ [9], where *c* and *B* represent the speed of light and the bandwidth, respectively. Thus, the range resolution for each mode is 10 cm and 5 cm, respectively. In the case of short-range detection, a wider bandwidth is used to detect nearby objects in high resolution, which means that one object can be detected as more points.

**Figure 3:**
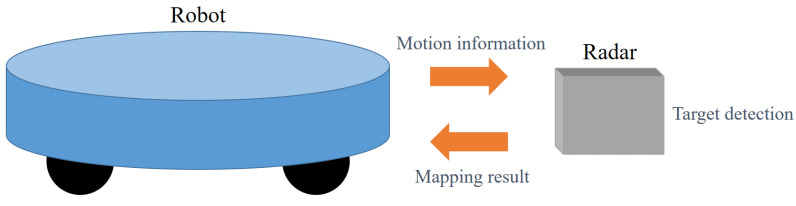
Information exchanged between the robot and the radar.

This FMCW radar sensor is mounted on the front of the robot as shown in Figure 3. First, the robot passes information related to the robot’s motion (e.g., the velocity and rotation angle of the robot) to the radar. This robot has a shape similar to a robot vacuum cleaner and can perform linear and rotational motions. Here, rotational motion means that the robot rotates at a constant angular velocity about the central axis. Then, the radar sensor estimates the distance, velocity, and angle information of targets located in the field of view (FOV). Finally, the radar sensor can map the surrounding environment combining the motion information and its own target detection results.

## 3. Radar Signal Analysis

In this section, a method of estimating the distance, velocity, and angle information of a target from data acquired by the dual-mode FMCW radar system is described. Using the FMCW radar signal shown in Figure 2, the relative distance to the target and the relative velocity of the target can be estimated. In addition, using the multiple antenna elements in Figure 1b, the angle information between the radar sensor and the target can be estimated.

### 3.1. Distance and Velocity Estimation Using FMCW Radar Signals

**Figure 4:**
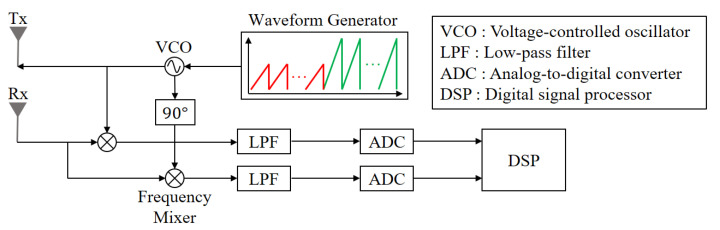
Block diagram of the FMCW radar.

The FMCW radar signal in Figure 2 is radiated through the transmit antenna and then reflected from the target. The received signal includes the time delay due to the relative distance to the target and the Doppler frequency due to the movement of the target. The received signal is converted to a baseband signal by passing through a frequency mixer and a low-pass filter. Then, the baseband signal is sampled in the time domain as it passes through an analog-to-digital converter, as shown in Figure 4.

**Figure 5:**
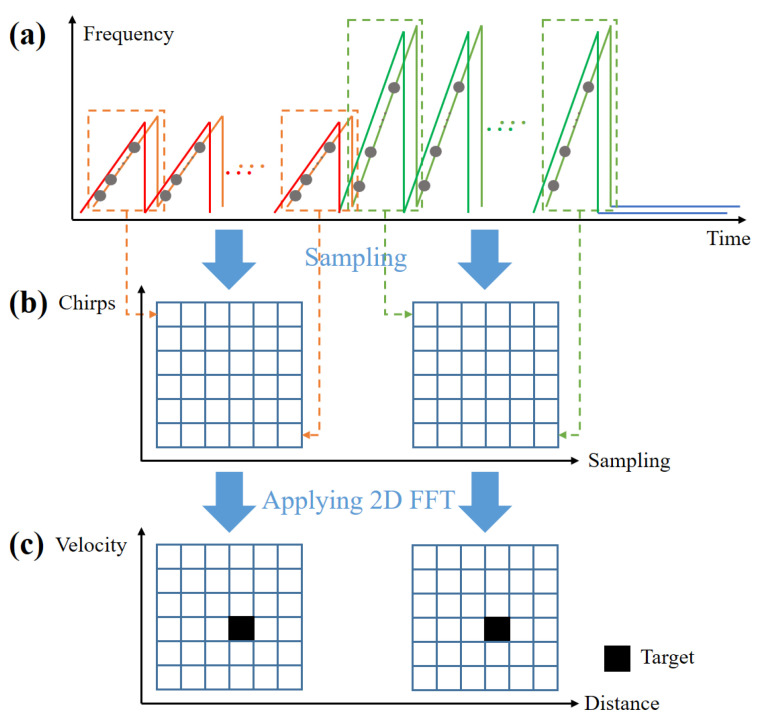
Distance and velocity estimation process in the FMCW radar system: (**a**) transmitted and received FMCW radar signals on the time-frequency axis; (**b**) the time-sampled signals in the form of the matrix; (**c**) 2D FFT results.

Then, the sampled baseband signal for the *l*-th chirp [17] can be expressed as

(1) $$\begin{array}{c} x\left[n,\;l\right]=A_{k}\exp\left[j2\pi \left\{\left(\frac{2B(R_{k}+v_{k}T_{c}l)}{T_{c}c}+\frac{2f_{c}v_{k}}{c}\right)nT_{s}+\frac{2f_{c}\left(R_{k}+v_{k}T_{c}l\right)}{c}\right\}\right], \end{array}$$

where k(k=1,2,⋯,K) is the index of the target and n(n=0,1,⋯,N−1) is the index of the time sample. In addition, $A_{k}$ indicates the amplitude of the baseband signal, and $R_{k}$ and $v_{k}$ represent the distance to the *k*-th target and the velocity of the *k*-th target, respectively. In addition, $T_{c}$ and $T_{s}$ denote the sweep time of each chirp and the sampling time, respectively. Then, the time-sampled signals for each chirp can be arranged in a matrix as shown in Figure 5b. In our radar system, $N_{L}$ and $N_{S}$ chirps are used in each long-range detection mode and short-range detection mode and *N* points are sampled for each single chirp, resulting in two matrices of size $N_{L}\times N$ and $N_{S}\times N$. If the fast Fourier transform (FFT) is applied on the sampling axis of the matrix, the matrix data are converted to data on the distance axis. In addition, if the FFT is applied on the chirp axis, it is converted into the data about the velocity axis [14]. In other words, by applying the 2D FFT to the sampled and rearranged FMCW radar signal, the relative distances to the targets and the relative velocities of the targets can be estimated at the same time [18], as shown in Figure 5c. Finally, target detection results in long-range mode and short-range mode are stored every frame.

### 3.2. Angle Estimation Using Multiple Antenna Elements

To accurately determine the position of a target, it is necessary to know not only the distance to the target but also the angle between the radar and the target. In general, the angular resolution improves as the number of antenna elements increases [11]. However, as the number of antenna elements increases, the size of the radar sensor also increases. Thus, to more accurately obtain the angle information of the target without significantly increasing the size of the radar sensor, we adopt the MIMO antenna system using multiple antenna elements.

In the short-range detection mode, one transmit antenna element and four receiving antenna elements (i.e., 1 × 4 single-input multiple-output (SIMO) antenna system) are used. In addition, the spacing between the receiving antenna elements is $d_{R}\times \left[e_{1},\;e_{2},\;e_{3}\right]$ in sequence, where $d_{R}$ denotes the basic unit of the spacing between receiving antenna elements. The antenna spacing can be determined in several ways, but we used the minimum-redundancy linear array that shows the maximum resolution for a given number of antenna elements by minimizing the number of redundant spacing in the array [19]. On the other hand, two transmit antenna elements and four receiving antenna elements (i.e., 2 × 4 MIMO antenna system) are used in the long-range detection mode. In this case, the spacing between the receiving antenna elements is the same as the short-range detection mode, and the spacing between the transmit antenna elements is set to $d_{T}=e_{t}d_{R}$, where $e_{t}$ is an integer value for the spacing between transmit antenna elements.

**Figure 6:**
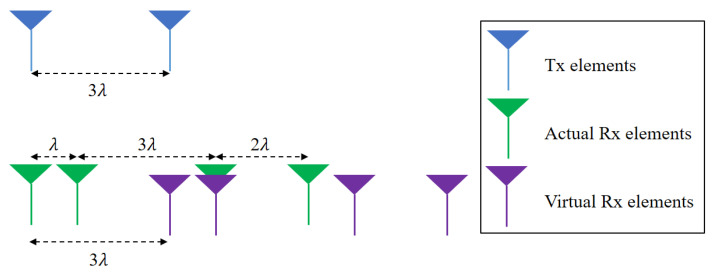
Expansion of receiving channels in the MIMO antenna system.

Assuming that the spacing between the antenna elements is very small compared to the distance between the radar and the target, almost parallel signals are received at each antenna element. Under this assumption, in the short-range detection mode, the phase values of the signals received from each antenna element become $\frac{2\pi \sin\theta_{k}}{\lambda }d_{R}\times \left[0,\;e_{1},\;e_{1}+e_{2},\;e_{1}+e_{2}+e_{3}\right]$, where $\theta_{k}$ is the angle between the radar and the *k*-th target and λ is the wavelength corresponding to the center frequency of the FMCW radar signal. Here, the phase value of the first receiving antenna element is set as a reference value, and λ becomes 4.8 mm because the radar system uses 62 GHz as the center frequency. In addition, the concept of the minimum redundancy linear array can be extended to the MIMO antenna system to achieve high angular resolution [20]. For long-range detection mode, because two transmit antenna elements with $d_{T}$ spacing are used, more antenna spacing combinations can be generated by the MIMO antenna principle [11]. Thus, the phase values of the signals received by each antenna element become $\frac{2\pi \sin\theta_{k}}{\lambda }d_{R}\times \left[0,\;e_{1},\;e_{1}+e_{2},\;e_{1}+e_{2}+e_{3},\;e_{t},\;e_{t}+e_{1},\;e_{t}+e_{1}+e_{2},\;e_{t}+e_{1}+e_{2}+e_{3}\right]$ in the long-range detection mode. Figure 6 shows the expansion of the number of receiving channels in the MIMO antenna system. Along with the actual receiving channels, virtual receiving channels are generated by the spacing between the transmit antenna elements. Because the time-division multiplexing scheme [21] is used in the transmission process, transmitted signals can be distinguished at the receiving antenna end.

In addition to this, we use an array interpolation technique to create more antenna spacing combinations. This method uses signals received from limited antenna elements and generates interpolated signals as if they were received from more antenna elements. In general, signals are interpolated using a transform matrix generated by the method of least squares [12]. We also use the linear least squares (LLS) to create more antenna spacing combinations in the SIMO and MIMO antenna systems [22]. The transformation matrix is not created every time a signal is received from the antenna but is predetermined by setting the FOV and the spacing to be interpolated. Thus, the transform matrix once calculated from the LLS method can be used continuously without changes in the radar system. In our radar system, the empty antenna spacing between 0 to $d_{R}\times \left(e_{1}+e_{2}+e_{3}\right)$ is interpolated at $d_{R}$ spacing in short-range detection mode. Even in long-range detection mode, the empty antenna spacing between 0 to $d_{R}\times \left(e_{t}+e_{1}+e_{2}+e_{3}\right)$ is interpolated at $d_{R}$ spacing.

**Figure 7:**
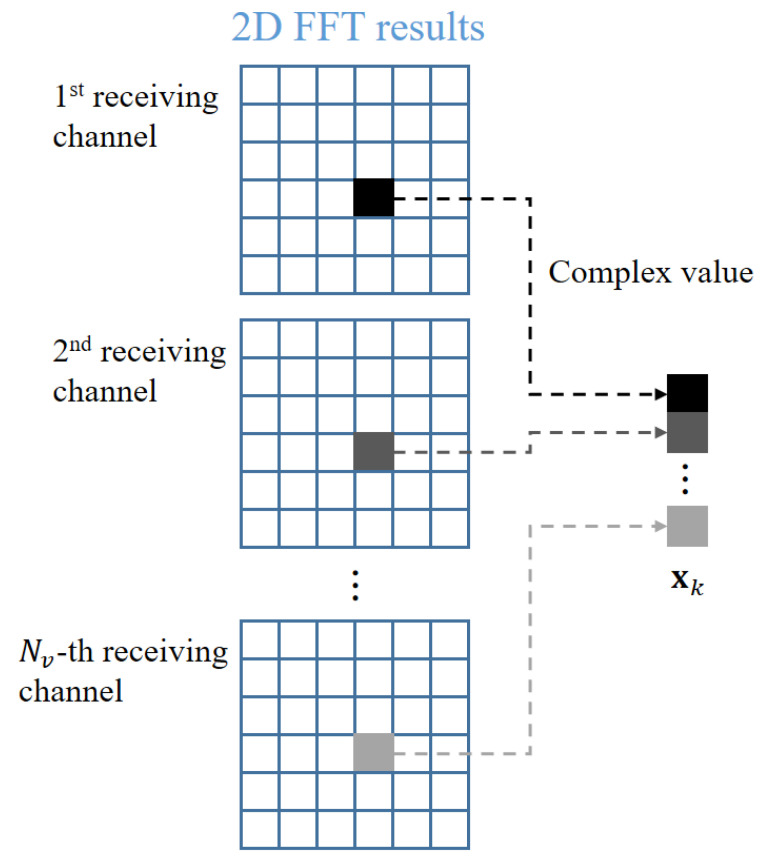
Angle estimation process in the FMCW radar system.

Finally, the angle of the target is estimated by finding the phase difference values of the received signals. For example, angle estimation algorithms, such as the conventional beamformer (i.e., Bartlett beamformer) [23], minimum variance distortionless beamformer (i.e., Capon beamformer), [24], multiple signal classification (MUSIC) [25], and estimation of signal parameters via rotational invariance techniques (ESPRIT) [26], can be used to estimate the phase difference. In general, these angle estimation algorithms can be applied directly to the time-sampled signals of each receiving channel [27]. In that case, we can estimate the incident angles of multiple signals received at the same time. However, it is difficult to pair the estimated angle values with the targets existing in the 2D FFT domain of Figure 5c. Thus, we use the angle estimation in the 2D FFT domain that accurately extracts only the angle information of each target [28], as shown in Figure 7. After 2D FFT of the signals received from each receiving antenna element, the values corresponding to the *k*-th target are collected and generated as a vector $x_{k}$. Because the distance and velocity information acquired from each receiving antenna channel is equivalent for the same target, the signal values at the same indices from the 2D FFT results are selected. Basically, because the FFT is a linear transformation, the angle information of the target is included in the phase of the frequency-domain signal even after the 2D FFT. In other words, $x_{k}$ still contains the values of the phase difference between each receiving channel. Finally, we create the correlation matrix using $x_{k}$, which can be expressed as

(2) $$\begin{array}{c} R_{k}=\frac{1}{N_{v}}x_{k}x_{k}^{H}, \end{array}$$

where $N_{v}$ is the number of receiving channels extended by the MIMO antenna principle and the array interpolation. This correlation matrix can be used in the spectrum-based angle estimation algorithms, such as the Bartlett and Capon, and the subspace-based angle estimation algorithms, such as the ESPRIT and the MUSIC. In this study, the Bartlett beamformer is used to estimate the angle of the target because it requires less computation than other algorithms [29] and exhibits stable performance even with coherent sources [30]. The Bartlett beamformer can be expressed as

(3) $$\begin{array}{c} P\left(\theta \right)=a^{H}\left(\theta \right)R_{k}a\left(\theta \right), \end{array}$$

where a(θ) denotes the steering vector [27]. Therefore, the angle θ that makes the value of P(θ) maximize becomes the estimated angle of the *k*-th target.

Finally, the specifications for each detection mode are summarized in Table 1. The maximum detectable range is determined by the antenna beam pattern and transmit power, and each mode uses the same transmit power. Thus, in the long-range detection mode, because the antenna beam pattern was designed to detect objects up to 20 m with the same transmit power, it inevitably has a narrow FOV. On the other hand, in the short-range detection mode, the antenna beam pattern was designed to detect objects up to 10 m with the same transmit power, so it has a wider FOV.

### 3.3. Detection Results in Dual-Mode

**Figure 8:**
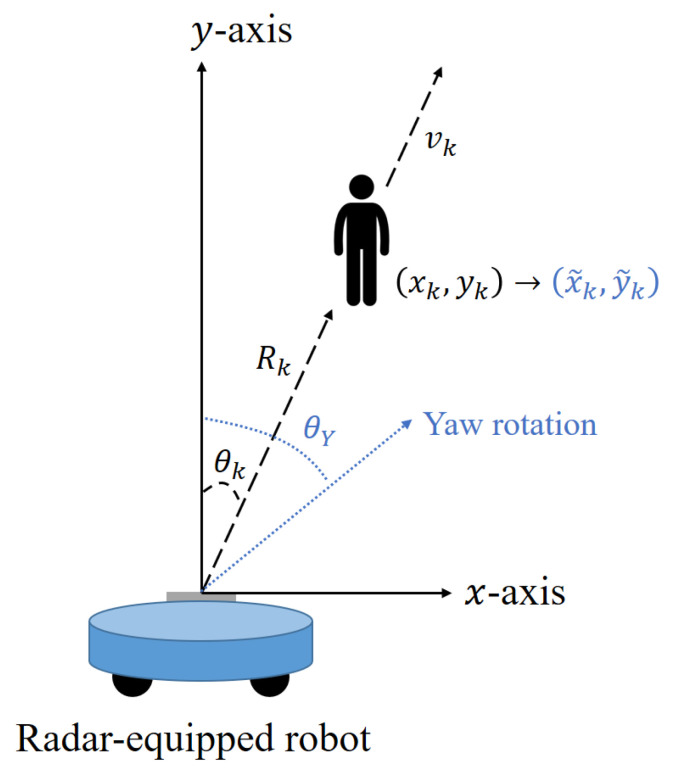
Representation of target coordinates in a 2D range map.

The distance obtained in Section 3.1 is the distance in the radial direction to the target based on the position of the radar-equipped robot, and the angle obtained in Section 3.2 is the angle between the front direction of the radar and the target, as shown in Figure 8. Therefore, the distance and angle information of the *k*-th target can be converted into the 2D position coordinates based on the radar position, which is expressed as

(4) $$\begin{array}{c} \left(x_{k},\;y_{k}\right)=\left(R_{k}\cos\theta_{k},\;R_{k}\sin\theta_{k}\right). \end{array}$$

In addition, a radar-equipped robot performs not only straight movement, but also circular rotation. Thus, to draw a 2D range map while accumulating the coordinates of the detected targets, the yaw rate of the robot must also be considered. If the robot performs yaw rotation by the angle $\theta_{Y}$, the position of the *k*-th target in Equation (4) will be changed as follows:

(5) $$\begin{array}{c} \left({\tilde{x}}_{k},\;{\tilde{y}}_{k}\right)=\left(R_{k}\cos\left(\theta_{Y}-\theta_{k}\right),\;R_{k}\sin\left(\theta_{Y}-\theta_{k}\right)\right). \end{array}$$

Therefore, if the detection results are accumulated without considering the yaw rate, the accuracy of the mapping becomes very low. The yaw rate of the robot can be obtained from its motor, and the detection result must be corrected every frame using this value.

**Figure 9:**
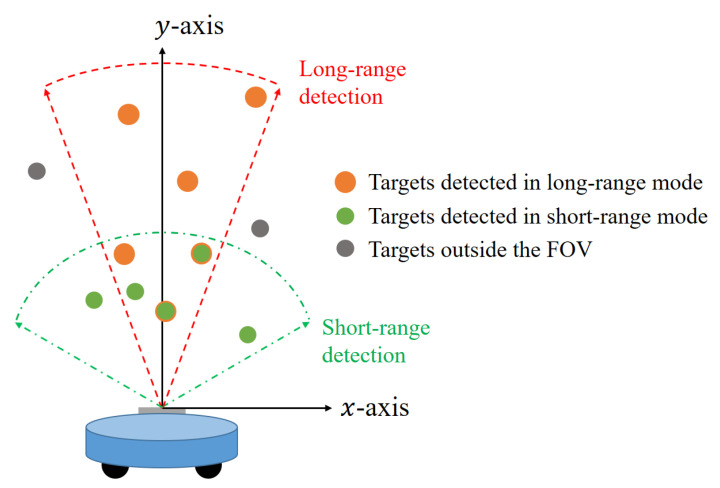
Combination of detection results from both modes.

These 2D target detection results can be obtained in each of the two modes and they are finally combined, as shown in Figure 9. The detection result of the short-range detection mode is superimposed on that of the long-range detection mode. In the case of targets detected in both modes, the reliability of the detection result is high. However, in the case of a target detected only in one mode, the reliability may not be high. In the case of such targets, they can be removed through the target tracking. In addition, because the robot can perform circular rotation, targets outside the FOV can also be detected through its rotational motion.

## 4. Indoor Environment Mapping Using Dual-Mode Radar Detection Results

**Figure 10:**
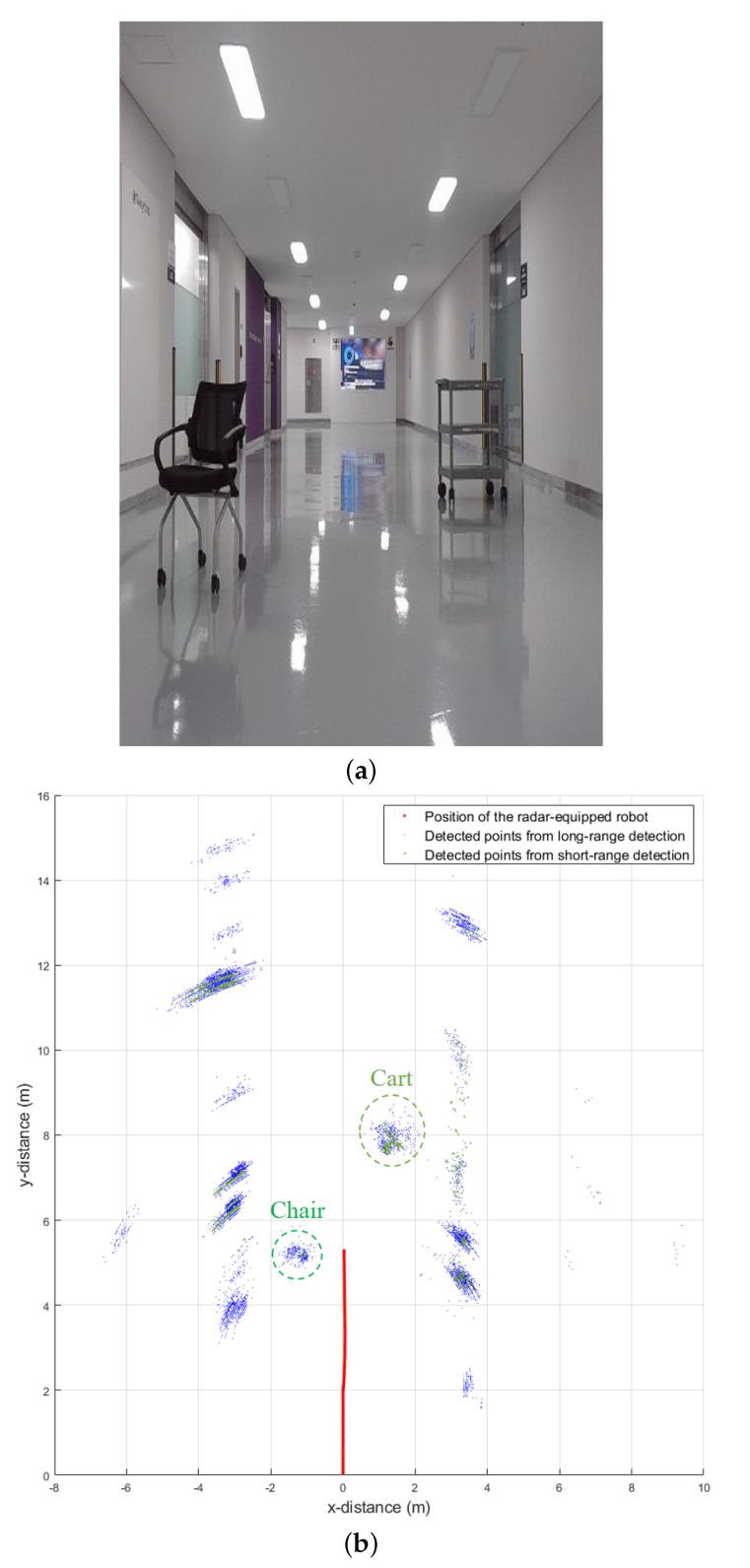
Measurement in an indoor environment: (**a**) a photograph of the environment; (**b**) accumulated detection results of the dual-mode radar.

After mounting the radar on the robot, we accumulated radar signal data in the indoor environment. The radar-equipped robot starts driving straight in the corridor of the building, as shown in Figure 10a. In the initial driving environment, a chair and a cart of different sizes are located at different distances. In our radar system, one frame is set to 50 ms, which implies that the radar sensor derives the target detection results 20 times per second. In addition, because we use 1.5 GHz and 3 GHz as bandwidths in each mode, the range resolution becomes 10 cm and 5 cm, respectively. That is, even if the size of objects existing indoors is quite small, it can be detected as several points. Figure 10b shows the radar detection results accumulated for 500 frames in this environment, in which we can see that the chair and the cart are detected as multiple points. In this figure, the detected points in each mode are represented in different colors. Because these three-dimensional objects, such as the chair and the cart, have different radar cross sections for each part, it is difficult to derive the exact size of the object. However, through repeated radar detection, we can determine the approximate size of the object. In this process, detected points for each object can be grouped through a clustering algorithm such as the density-based spatial clustering of applications with noise [31].

**Figure 11:**
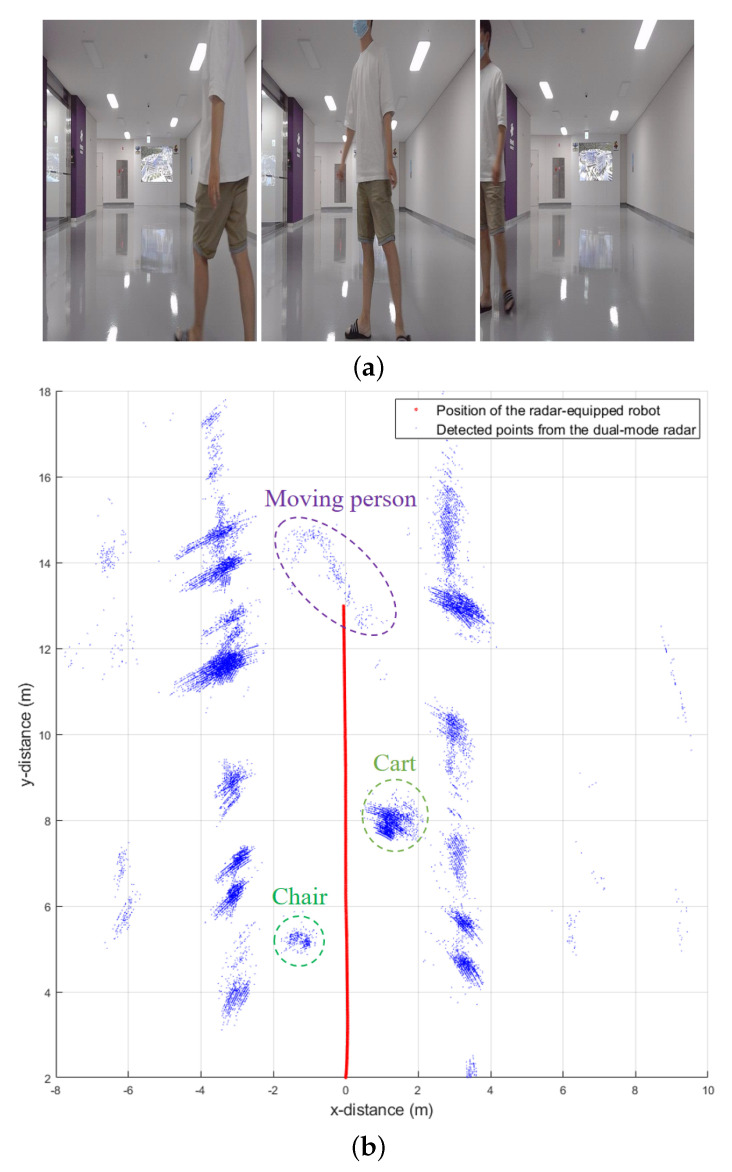
Measurement in an indoor environment: (**a**) photographs of the environment; (**b**) accumulated detection results of the dual-mode radar.

Then, the radar-equipped vehicle keeps moving forward and encounters a person walking from right to left, as shown in Figure 11a. In this experiment, a man passed the radar’s FOV at a speed of about 1 m/s. In Figure 11b, radar detection results for additional 1000 frames were accumulated and drawn. Looking at the figure, the moving person is also detected as multiple points in the radar detection results. However, compared to a chair or a cart, the number of detected points is fewer because the person has a smaller radar cross section and the width of the human body is shorter.

**Figure 12:**
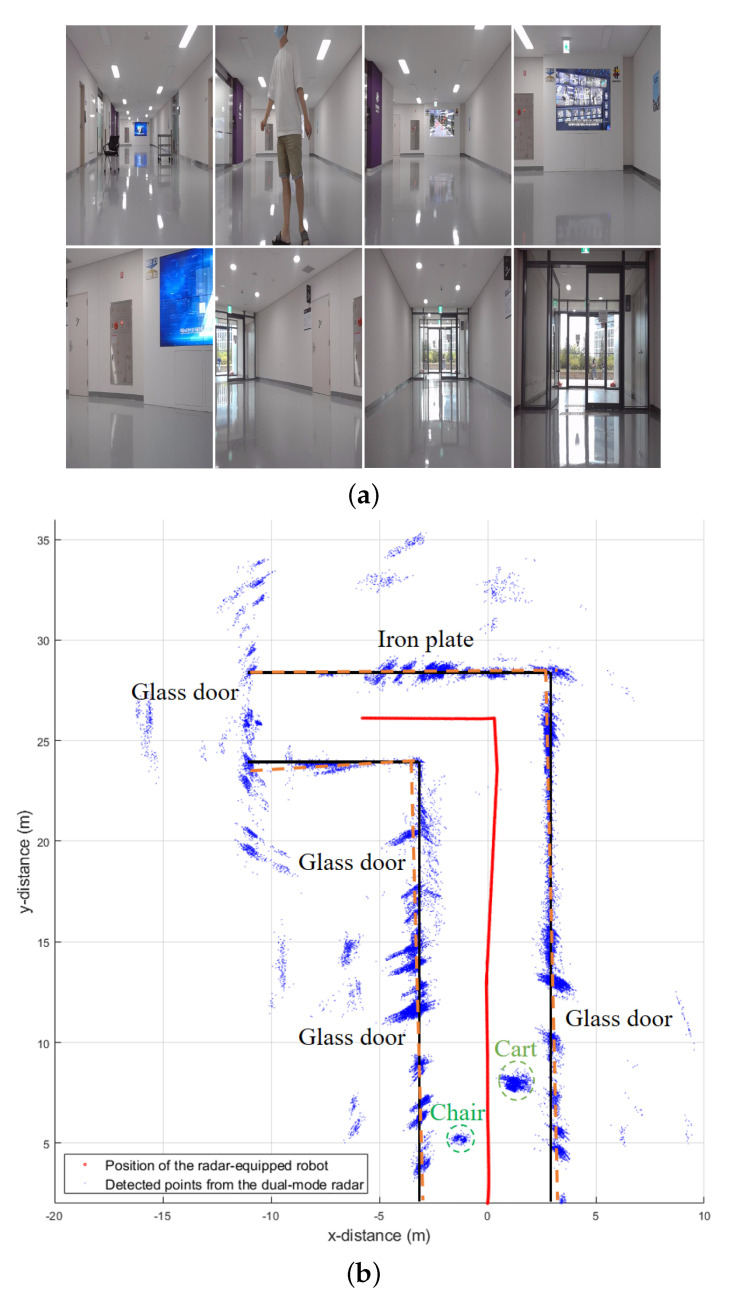
Indoor environment mapping using dual-mode radar detection results: (**a**) photographs of the environment; (**b**) final mapping result after removing moving targets.

To accurately draw a map around a robot using radar sensor data, the points corresponding to the moving target must be removed. In this study, we use a method to identify moving targets by using the relative velocity between the detected points and the radar-equipped robot. This method can be applied without high computational complexity because radar sensors can directly extract target velocity information, unlike sensors such as lidars and cameras. If the velocity of the robot received from the motor is $v_{M}$ and the index of the target detected in dual-mode in each frame is $\tilde{k}$, the stationary target can be selected by finding all $\tilde{k}$ that satisfies

(6) $$\begin{array}{c} \left|v_{M}-v_{\tilde{k}}\right|<\epsilon , \end{array}$$

where ϵ is the threshold value. Through this method, the moving velocity of the robot is compared with the velocities of the detected targets. Even if the radar does not receive the moving velocity of the robot from the motor, a method of estimating the velocity of the robot using only the radar sensor data was proposed in [32]. Finally, Figure 12b shows the final mapping result after removing detected points corresponding to moving targets, and the points caused by the moving person almost disappeared. In Figure 12b, because most of the radio waves are not reflected on the glass door and pass through them, the detected points for those doors are less visible in the mapping results. In addition, points detected outside the wall can be considered ghost targets generated by multiple propagation paths of radio waves.

To evaluate the accuracy of the position estimation, we used the 2D floor plan of the building. In other words, the actual map data of the indoor environment drawn with black lines in the figure were compared with the detection results from the radar sensor data. To verify how similar the actual wall position and the wall estimated from the detected points were, the trend lines for each wall were estimated by the random sample consensus (RANSAC) [33] algorithm. The RANSAC algorithm can automatically extract the trend line for points on the plane. We applied the RANSAC method to the points detected on each of four walls, and the results are indicated by orange dashed lines in Figure 12b. As shown in the figure, the trend lines predicted by the RANSAC were very similar to the actual wall positions, and had a slope error within three degrees. In addition, we compared the actual and the estimated positions of the robot itself. The robot’s position was estimated from motion information acquired from the robot’s motor. In our measurement, the radar-equipped robot was set to move in a straight line, but the estimated positions are slightly off the straight line, as shown in the figure. Finally, the actual and the estimated positions of the robot itself showed a mean absolute error of 5%.

## 5. Conclusions

In this paper, we presented the mapping results based on the data acquired from the dual-mode radar sensor. The radar sensor we designed can alternately transmit two different waveforms that use different bandwidths in each frame, allowing short-range detection and long-range detection at the same time. This radar system has excellent range resolution due to its high center frequency and wide bandwidth, which enables it to detect one object as multiple points. In addition, we used array interpolation with the MIMO antenna system to improve the angular resolution. After mounting this radar on a small robot, radar sensor data were acquired while driving in an indoor corridor environment. Finally, by combining the motion information received from the robot with the radar detection results, the radar-equipped robot mapped the indoor corridor environment while estimating its position. In addition, a map with improved accuracy for the indoor environment was obtained when we used the velocity information of moving targets extracted from the radar sensor data. To increase the accuracy of radar-based mapping, it can be effective to use map data generated from existing radar detection results as a priori information. The radar sensor-based SLAM is expected to be used effectively in an environment where camera or laser sensor-based SLAM performance is degraded.

## Figures and Tables

**Table 1:** Specifications for each detection mode.

Detection Mode	Long-Range Mode	Short-Range Mode
Bandwidth, *B* (GHz)	1.5	3
The number of chirps, $N_{L}$ and $N_{S}$	256	256
The number of time samples, *N*	128	128
Maximum detectable range (m)	20	10
Range resolution (m)	0.1	0.05
Maximum detectable velocity (m/s)	8	8
Velocity resolution (m/s)	0.315	0.315
FOV (deg.)	−20∼20	−60∼60
Transmit power (dBm)	10	10
Total transmission time (ms)	50	

## Data Availability

Data sharing not applicable.

## Abbreviations

The following abbreviations are used in this manuscript:

2D	two-dimensional
ESPRIT	Estimation of signal parameters via rotational invariance techniques
FFT	Fast Fourier transform
FHR	Fraunhofer Institute for High Frequency Physics and Radar Techniques
FMCW	Frequency-modulated continuous wave
FOV	Field of view
MIMO	Multiple-input multiple-output
MUSIC	Multiple signal classification
PCB	Printed circuit board
RANSAC	Random sample consensus
SIMO	Single-input multiple-output
SLAM	Simultaneous localization and mapping
